# Statistical Shape Modelling the In Vivo Location of Acetabular Wear in Retrieved Hip Implants

**DOI:** 10.3390/bioengineering10010046

**Published:** 2022-12-29

**Authors:** Sean Bergiers, Johann Henckel, Harry Hothi, Anna Di Laura, Chris Goddard, David Raymont, Furqan Ullah, Ross Cotton, Rebecca Bryan, Alister Hart

**Affiliations:** 1 Institute of Orthopaedics and Musculoskeletal Science, University College London, London WC1E 6BT, UK; 2 Royal National Orthopaedic Hospital, Stanmore HA7 4LP, UK; 3 Synopsys Northern Europe Ltd., Exeter EX4 3PL, UK

**Keywords:** statistical shape modelling, retrieval analysis, hip replacement, CT imaging

## Abstract

Edge-wear in acetabular cups is known to be correlated with greater volumes of material loss; the location of this wear pattern in vivo is less understood. Statistical shape modelling (SSM) may provide further insight into this. This study aimed to identify the most common locations of wear in vivo, by combining CT imaging, retrieval analysis and SMM. Shape variance was described in 20 retrieved metal-on-metal acetabular surfaces. These were revised after a mean of 90 months, from 13 female and seven male patients. They were positioned with a mean inclination and anteversion of 53° and 30°, respectively. Their orientation, in vivo, was established using their stabilising fins, visible in pre-revision CT imaging. The impact of wear volume, positioning, time, gender and size on the in vivo location of wear was investigated. These surfaces had a mean wear volume of 49.63 mm^3^. The mean acetabular surface displayed superior edge-wear centred 7° within the posterosuperior quadrant, while more of the volumetric wear occurred in the anterosuperior quadrant. Components with higher inclination had greater superior edge-wear scars, while a relationship was observed between greater anteversion angles and more posterosuperior edge-wear. This SSM method can further our understanding of hip implant function, informing future design and may help to refine the safe zone for implant positioning.

## 1. Introduction

Mechanical wear at the bearing surface of hip replacements can influence their clinical performance, resulting in impaired function and the release of harmful debris. Following the large-scale failure of metal-on-metal (MOM) hips, numerous retrieval studies have used standardised methodologies to investigate the extent of wear on these surfaces [1,2,3,4,5,6]. The resulting bearing wear volumes and maps informed the clinical use of blood metal ion levels as an indicator of failing hips, as well as identifying an association between wear at the edge of the acetabular cup and high volumes of wear debris [7].

Although MOM implants are scarcely used at present, their analysis provides an invaluable insight into the mechanics of hip replacements. Whilst it is known that acetabular edge wear occurs in vivo, its orientation within the acetabular cavity is less understood. A recent study combined retrieval metrology with postoperative 3D computed tomography (CT) to locate acetabular edge-wear in vivo [8]. This ability to orientate acetabular wear maps within the patient provides a unique opportunity to investigate the relationship between the location of wear and different surgeon, implant and patient (SIP) factors; however, a more encompassing approach is required to fully understand the most prominent locations of acetabular wear. 

Statistical shape models (SSM) are used to describe the shape and pose of a population of related geometries. They can be particularly useful in analysing anatomical features, allowing the mean shape and shape variance within a cohort to be visualised [9]. In orthopaedics, SSM has been implemented to describe populations of pelvises, in order to classify large acetabular defects and reconstruct their original centre of rotation, informing the placement and design of restorative implants [10,11,12,13]. Its application to the surfaces of retrieved acetabular hip components would allow wear patterns across their entire surface, from a wide cohort of patients, to be visualised collectively in single map.

The overarching aim of this study was to identify the most common in vivo wear patterns present in the acetabular component of hip replacements, using a novel method of combining CT imaging and retrieval analysis techniques to generate a statistical shape model. 

## 2. Materials and Methods

**Figure 1:**
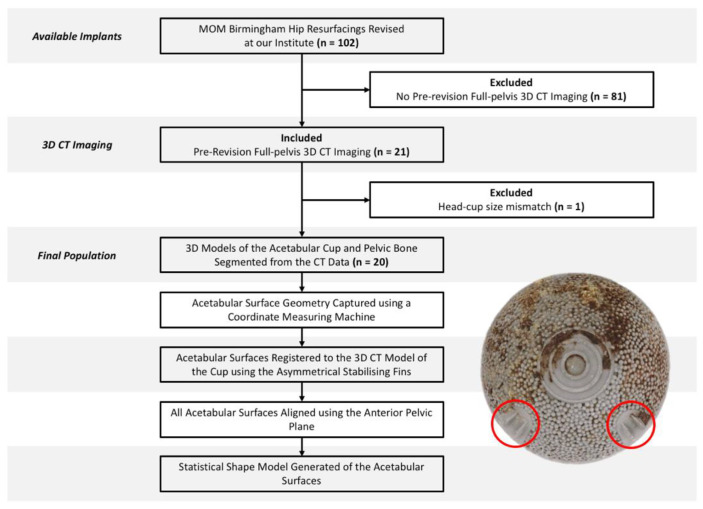
Study design and workflow, accompanied by the backside surface of a Birmingham acetabular component and its asymmetrical stabilising fins (circled in red).

The acetabular component of twenty retrieved MOM Birmingham hip replacements (BHR, Smith and Nephew, London, UK) were included in this study, which were composed of a cobalt-chrome-molybdenum alloy (CoCrMo). This implant design was specifically selected due to the asymmetric stabilising fins found on its backside surface. These implants were selected from a larger population of 102 BHRs, based on the inclusion criteria that pre-revision 3D CT images were available, capturing the pelvic bone and the implant prior to removal (Figure 1).

All twenty BHR implants were revised by two of the authors, following a mean time in vivo of 90 months (SD = 47), from 13 female and 7 male patients. Revision surgery was undertaken due to either an adverse reaction to metal debris (ARMD) (n = 9), unexplained pain (n = 8) or aseptic loosening (n = 3). The median size (external diameter) of their acetabular cups was 54 mm, ranging from 48 mm to 62 mm.

The in vivo position of each BHR was calculated through a bespoke software solution (Robin’s 3D; Robin Richards, London, UK), which used the anterior pelvic plane (APP) as a standardised coordinate system and reported values of anatomical inclination and anteversion.

### 2.1. Geometric Analysis of the Acetabular Cups

The articulating surface geometry of each retrieved cup was captured in the form of a point cloud, using a coordinate measuring machine (CMM, Carl Zeiss Ltd., Rugby, UK). Its 3 mm ruby stylus was instructed to capture up to 140,000 data points across their surfaces, along meridians of longitude that emanated at their poles and terminated within 1 mm of their edge [14,15]. The adopted scanning strategy utilised a point pitch (0.1 mm) to optimise the triangulation accuracy of the final surfaces. In each case, the span of both stabilising fin pairs was also captured through additional points recorded at the rim.

The volume of material loss from each acetabular surface was calculated using a previously validated, automated software solution [16,17]. The data points collected at the cup rim were excluded from this analysis. The centre coordinates and radius of the unworn sphere calculated for each component were also recorded for later use.

### 2.2. Registration of Articular Surfaces to CT Images 

**Figure 2:**
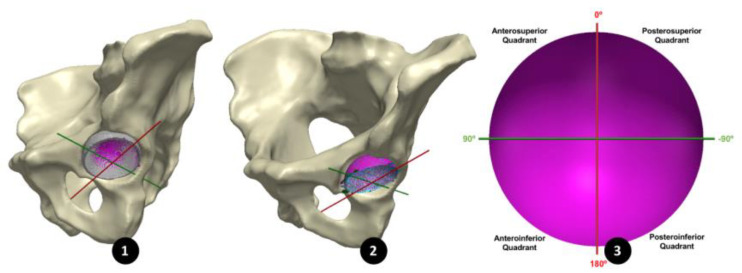
(1) An acetabular surface (purple) registered to the 3D model of its acetabular cup (grey), segmented form its 3D CT images. (2) All 20 acetabular surfaces registered using the CAPP axis, maintaining their in vivo orientation. (3) The CAPP axis (red) used to divide the acetabular surface into four quadrants.

Pre-revision CT images of the BHR implants, in vivo, were imported as Digital Imaging and Communications in Medicine (DICOM) files into Simpleware™ (Synopsys, Inc., Mountain View, CA, USA). Within this software, both implant and pelvic bone models were generated through a semi-automated segmentation process of thresholding. Minimal postprocessing was applied to reduce the presence of metal artifacts, while retaining geometrical accuracy. In order to isolate the acetabular cup from the BHR implant model, a sphere was best-fit to the femoral head component and subtracted through a Boolean operation. A plane was then best-fit to the cup rim to remove the relatively inferior portion of the BHR model, including the remanence of the femoral peg. Open surface representations of the acetabular cups, generated from the CMM data, were imported through Simpleware^TM^ as Stereolithography (STL) files and registered to the isolated cup model, using a semi-automated function to align their stabilising fins (Figure 2). Once registered, the acetabular cup surfaces and bone models were mirrored and scaled with respect to a 52 mm, left sided BHR implant.

### 2.3. Registration of the BHR surfaces using the Anterior Pelvic Plane

**Figure 3:**
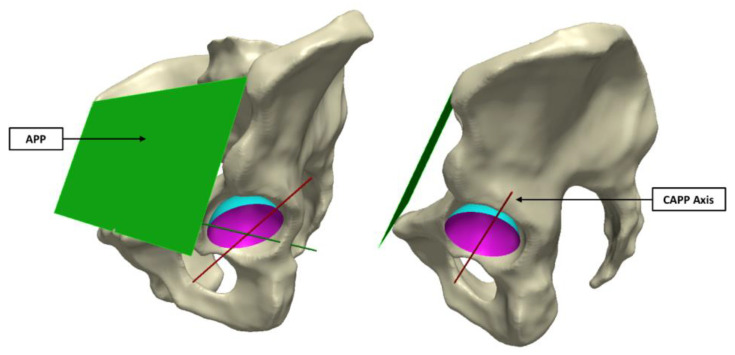
The CAPP axis was defined using a plane parallel to the APP (**left** image), which intersected the centre of the sphere formed by the acetabular surface (**right** image).

In order to align all 20 acetabular surfaces, while maintaining their in vivo orientation, a standardised coordinate system was defined using a plane parallel to the anterior pelvic plane (APP) that intersected the centre of the cup surface (Figure 3). As in a study by Bergiers et al. [8], this plane was termed the Cup-APP (CAPP) and was representative of the vertical standing position due to its relationship to the APP. The CAPP and its normal plane at the cup centre formed the vertical and horizontal axis of a new coordinate system in the cup rim plane, with the cup vector defining the final axes. These would also become the measurement axes from which the in vivo location of the primary wear scar would be determined, splitting the acetabular surface into four quadrants (anterosuperior, anteroinferior, posterosuperior and posteroinferior; Figure 2). 

### 2.4. Statistical Shape Modelling

The aligned acetabular surfaces were clipped at the cup-rim transition and capped to form closed surfaces. Each of these surfaces was discretised to form dense point sets that were mapped to a reference geometry, defined as the unworn sphere of the acetabular surface to which all other surfaces were scaled. Within Simpleware^TM^ a principal component analysis (PCA) was performed to generate a shape model that could be morphed from the mean acetabular surface geometry through the identified modes of variation (principal components). These modes were eigenvectors that describe the shape variance within the population, and were ranked based on the degree of variance in their direction.

Surface deviation maps were generated to compare the mean acetabular surface model with the reference unworn sphere, which would present the location of wear (wear map). A deviation above 5 microns from the unworn sphere was considered wear, as any deviation below was within manufacturing tolerances [18]; the minimum measurable wear volume was 0.14 mm^3^. The same approach was adopted to investigate the dominant modes of variance within this population of acetabular surfaces. Using an orthogonal view of the acetabular cup rim, the angular range of the primary wear scar was measured with respect to the vertical CAPP axis, allowing its centre to be defined. Anteriorly centred wear scars were represented by positive angles, while posteriorly centred scars were represented by negative angles. 

A leave-one-out study was performed to evaluate the contribution of each acetabular surface to the statistical shape model. In turn, each surface was excluded from the PCA, and the centre of the primary wear scar was measured in each mean model.

### 2.5. Impact of Surgeon, Implant and Patient Factors

In order to investigate the influence of volumetric material loss, inclination, anteversion, component size, gender and time to revision on the in vivo location of acetabular wear, further PCA’s were performed. These were conducted on a subset of the population, where the 20 acetabular surfaces were divided into two opposing groups for comparison. For example, to investigate the relationship between volumetric material loss and the in vivo location of wear, the surfaces were divided into high and low wearing implants based on whether they presented more than 2 mm^3^ of material loss. The influence of component positioning was investigated by dividing the surfaces into well- and mal-positioned groups, based on Lewinnek’s safe zone [19]. The sampling criteria for all variables in this investigation can be seen in Table 1. The mean wear map and the angular location of the primary wear scar were compared for both subgroups.

In order to validate the surface deviation maps generated in Simpleware^TM^ ScanIP, the same approach was adopted to assess the wear volume of each acetabular surface, in order to compare with the measurements performed by the previously validated software. A Bland–Altman analysis was undertaking reporting the mean error (SD) between the two volumes.

## 3. Results

The acetabular cups in this study had a mean volumetric material loss of 49.63 mm^3^ (standard deviation; SD = 141.2). These were positioned with mean inclination and anteversion angles of 53° (SD = 11) and 30° (SD = 17), respectively. Four cups were collectively well-positioned according to Lewinnek’s safe zone; nine had an inclination angle within Lewinnek’s criteria, while seven implants had a conforming degree of anteversion. 

**Figure 4:**
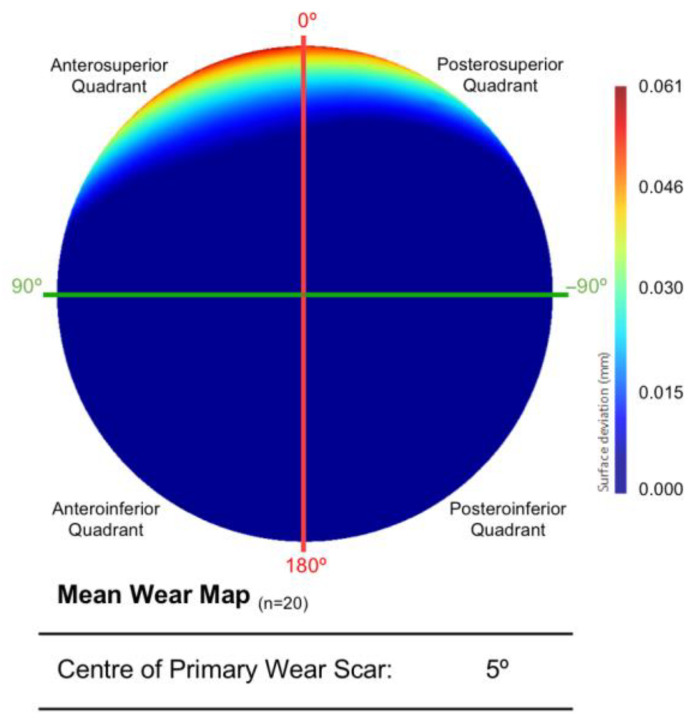
The mean acetabular surface generated through a PCA of the entire population (n = 20). This is presented as deviation map of their comparison with the as-manufactured reference surface, where the dark blue regions are considered unworn (mm).

The mean surface of all twenty acetabular surfaces generated during the first PCA can be seen in Figure 4, which is presented as a surface deviation map. The centre of its primary wear scar was located 5° into the anterosuperior quadrant.

**Figure 5:**
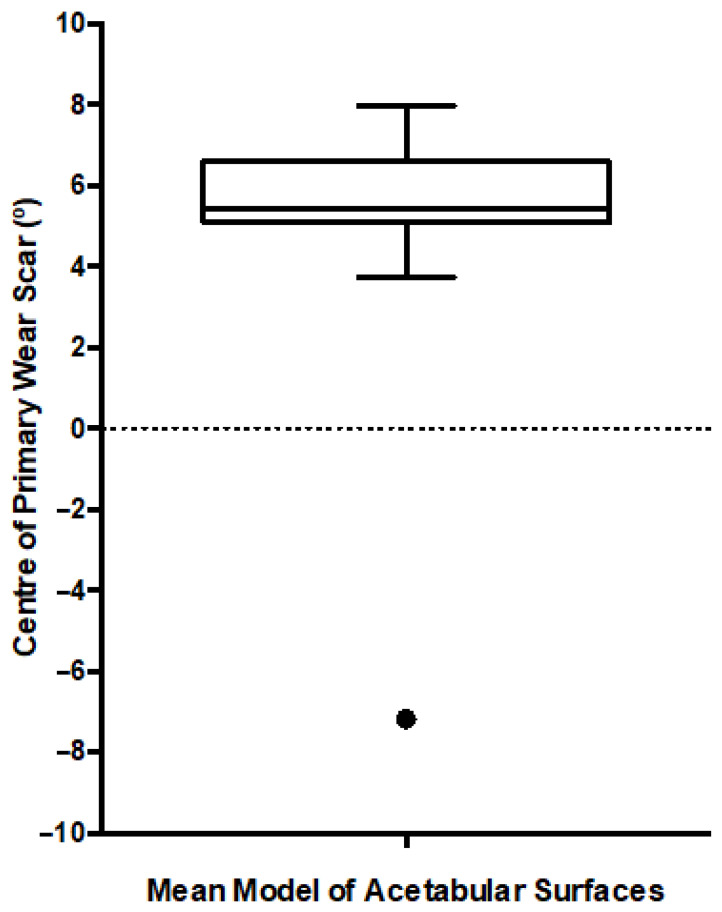
Box and Whiskers plot presenting the location of the primary wear scar centre, measured after each iteration of the leave-one-out study. The median is presented along with the range (min-max) and interquartile range. The black dot represents the single outlier.

The leave-on-out study conducted to assess the output from this first PCA found that a single acetabular surface significantly influenced the centre of the mean wear scar (Figure 5). The outlying surface was the highest wearing example in the population, surpassing its nearest neighbour by over 500 mm^3^. As a result, this acetabular surface was excluded from further analyses and the two groups were defined as detailed in Table 1.

**Figure 6:**
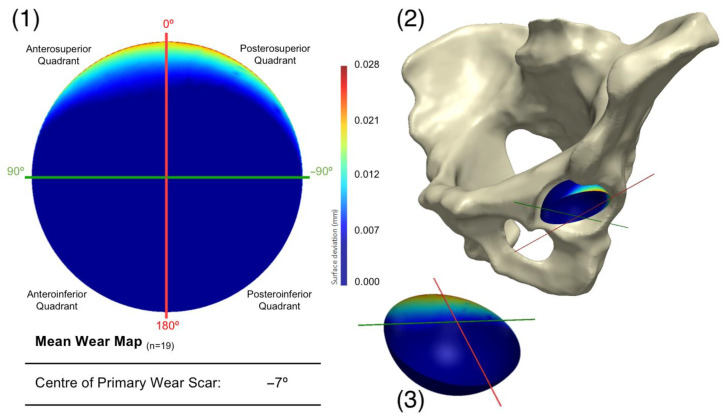
Presenting (1) the mean acetabular surface generated through a PCA of the population (n = 19), following the exclusion of a single surface. This is presented as deviation map of their comparison with the as-manufactured reference surface, where the dark blue regions are considered unworn. Its orientation within the pelvis is also presented (2), along with a 3D representation of the wear map (3).

Once the outlying acetabular surface was excluded, their mean geometry was regenerated through another PCA (Figure 6). The variance in shape within this population of acetabular surfaces could be described by 17 principal components. The centre of the primary wear scar was located 7° into the posterosuperior quadrant. Nevertheless, 5.73 mm^3^ of this material loss came from the anterosuperior quadrant, while 5.08 mm^3^ was in the posteroinferior quadrant. A further leave-one-out study was conducted to evaluate the influence of the remaining acetabular surface, which highlighted the weight carried by the highly worn examples in the population, as expected, and further exclusions was not deemed necessary.

**Figure 7:**
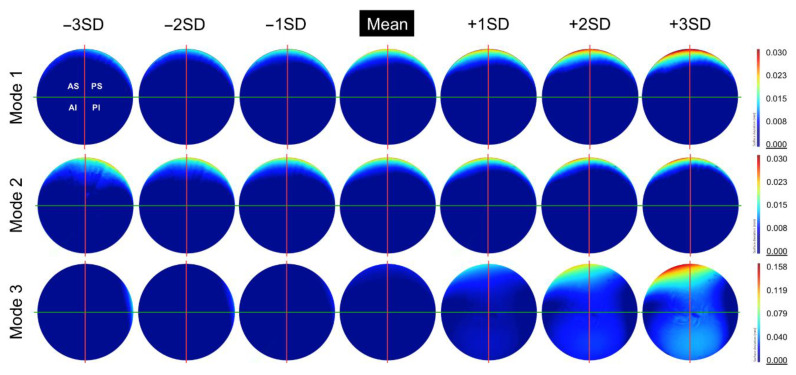
The change in geometry from −3SD to +3SD of the first 3 principal components (modes) calculated from this population of acetabular surfaces, presented as deviation maps of their comparison with the as-manufactured reference surface (mm).

The first three principal components were found to represent 89% of the variance within this population of acetabular surfaces, with 99% of the variance being described by the first 11 principal components. The change in geometry from −3SD to +3SD of the first three principal components is presented in Figure 7, as surface deviation maps. The first and most dominant mode of variance reflects an increase in linear depth at the primary wear scar. The concentration of wear to a smaller span of the acetabular edge is shown to be congruent with this trend, as well as a more anterior location. The second mode of variance presents the relationship between the location of the wear scar and its coverage towards the centre of the acetabular surface. A more posteriorly centred scar was found to be associated with greater areas of wear towards the acetabular centre. The third mode of variance appeared to describe the degree of pinching along the horizontal axis toward the one limit of the mode’s distribution, while pinching along vertical axis is represented by the opposing limit of the mode’s distribution. However, it was noted that the trend of increased pinching along the vertical axis was dampened somewhat by the presence of the primary wear scar.

### Impact of Surgeon, Implant and Patient Factors

**Figure 8:**
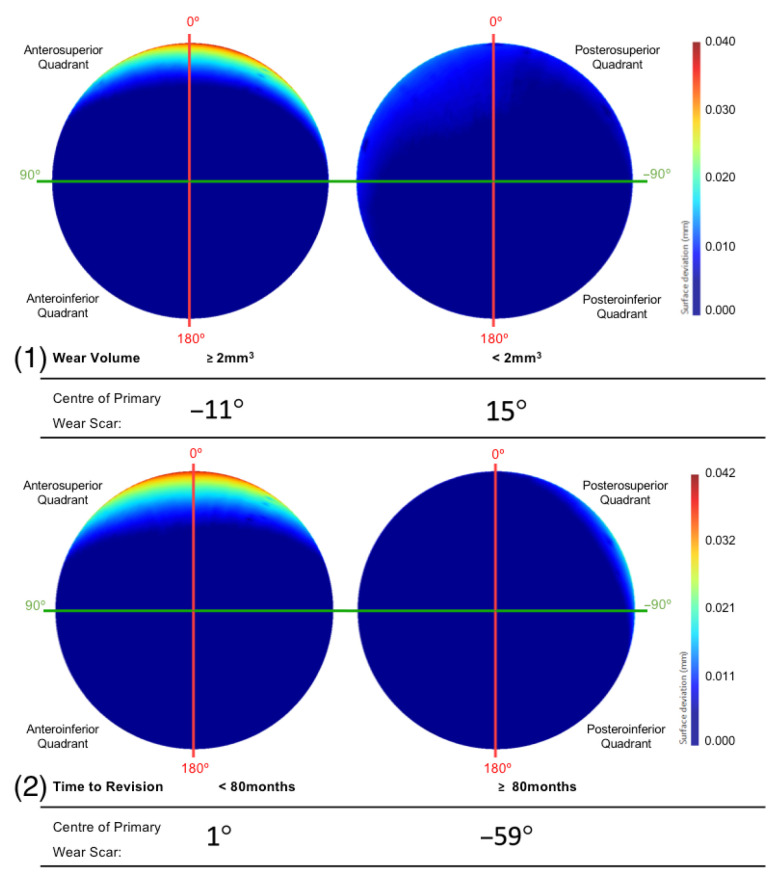
The mean surfaces generated following the subdivision of the acetabular surface, based on their (1) volumetric were and (2) time to revision. This is presented as deviation maps of their comparison with the as-manufactured reference surface (mm).

After dividing the population of acetabular surfaces into contrasting groups of volumetric wear, gender, position, time to revision and size, the mean geometry of each group was compared with respect to the location of their primary wear scar. The BHR acetabular surfaces that presented a wear volume in excess of 2 mm^3^ had a primary wear scar that centred about a position of 11°, while the low wearing examples had a mean wear scar centre of −15° (Figure 8). The implants that had remained in vivo for more than 80 months had a mean wear scar centre of −59°, while those that were revised prior to this time point had a mean wear scar centre of 1° (Figure 8).

**Figure 9:**
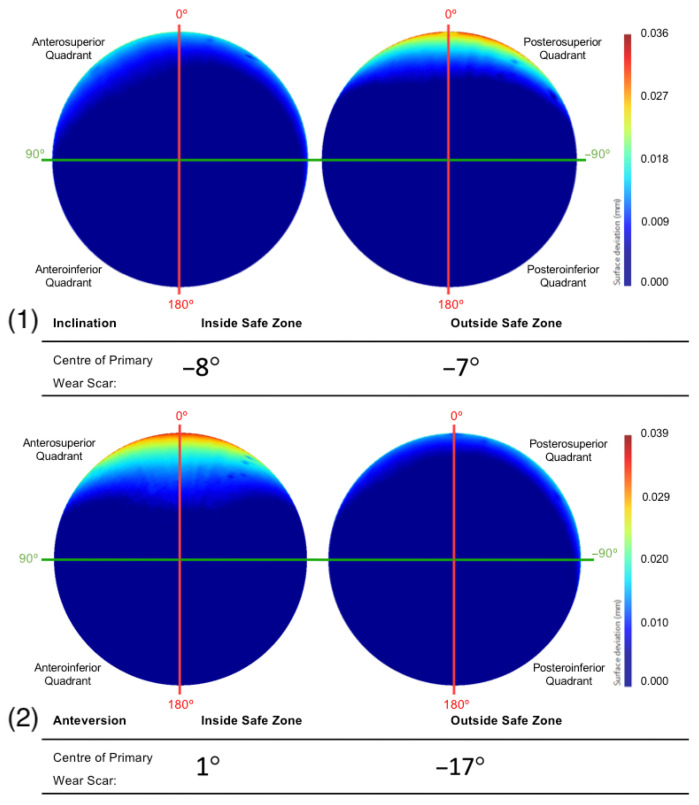
The mean surfaces generated following the subdivision of the acetabular surface, based on their (1) inclination and (2) anteversion angles. This is presented as deviation maps of their comparison with the as-manufactured reference surface (mm).

The primary wear scar centre of components that were positioned with an inclination angle within and outside Lewinnek’s safe zone was −8° and −7°, respectively (Figure 9). The centre of the primary wear scar of the components that were positioned with an anteversion angle within and outside Lewinnek’s safe zone was 1° and −17°, respectively (Figure 9).

**Figure 10:**
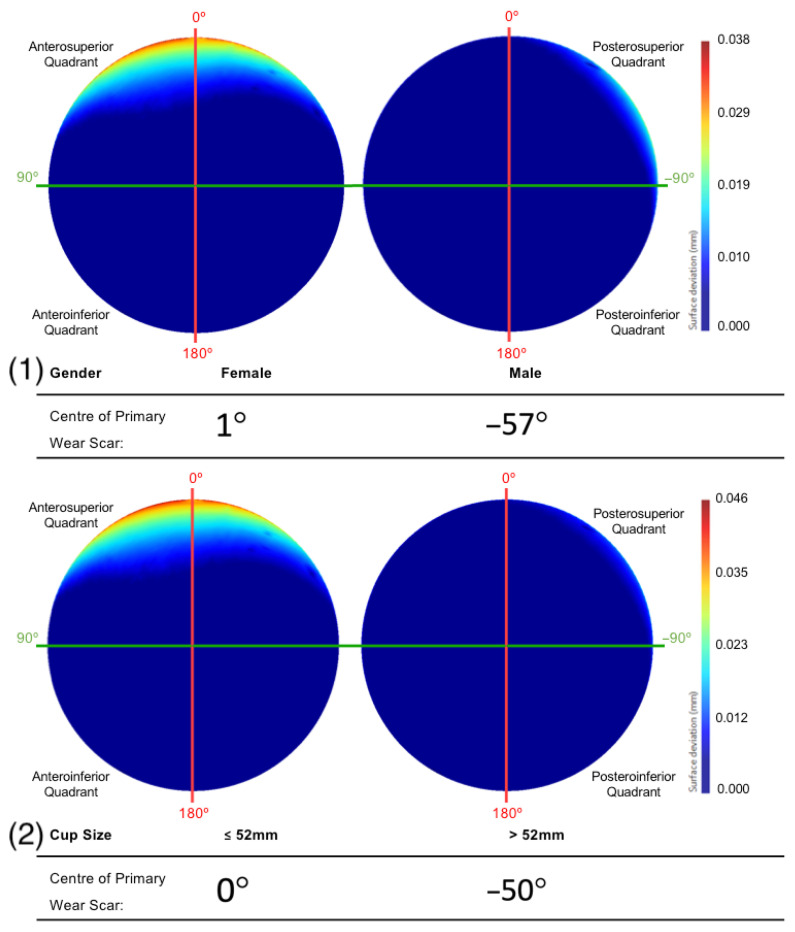
The mean surfaces generated following the subdivision of the acetabular surface, based on their (1) gender and (2) component size. This is presented as deviation maps of their comparison with the as-manufactured reference surface (mm).

Female patients had a mean primary wear scar centre of 1°, while male patients had mean wear scar centre of −56° (Figure 10). The implants that were sized 52 mm and below had a mean wear scar that was centred about a 0° angle, while the larger sizes had a wear scar centre of −50° (Figure 10).

Informed by the centre and radius of the implant’s unworn spheres calculated by the previously validated software, Simpleware Scan IP was able to replicate the material loss measurements presented above within a mean error of −0.60 mm^3^ (SD = 6.98). 

## 4. Discussion

This is the first study to apply statistical shape modelling to the analysis of retrieved orthopaedic implants. SSM was adopted to visualise and interpret shape variance within a population of retrieved resurfacing hips (BHR), with the objective of identifying the in vivo location of acetabular wear.

As the first to implement this methodology in this context, a practical and translatable approach had to be taken towards data interpretation. The mean model generated from the acetabular surfaces (n = 19) of these BHR hips were compared to an unworn (as-manufactured) reference surface, which was recreated using a previously validated and automated software solution [17]. The resulting surface deviation maps permitted the mean in vivo location of acetabular wear to be successfully identified. The primary wear scar found on this mean surface was located at the superior acetabular edge, where the articulating surface meets the rim of the implant, with virtually no other signs of surface damage. Its centre was measured 7° within the posterosuperior quadrant in vivo; however, the distribution of volumetric wear was subtly skewed towards the anteroposterior quadrant.

Such wear patterns are referred to as edge-wear, and are commonly found in acetabular cups during retrieval analysis [1,20,21,22]. This wear pattern has been correlated with elevated volumes of material loss, which may explain its prominence in the mean surface generated from the present population [5,23,24]. Deeper regions of wear outweighed subtler wear patterns, obscuring them in the mean deviation map. Many SIP factors have been associated with causing the bearing contact patch to approach the rim. These include a reduced diametrical clearance (difference in diameter between the cup and head components), a small arc of coverage angle (degree of coverage provided by the cup over the head) and a greater degree of inclination [3,25]. Although the mechanism behind edge-wear is widely understood, prior to the study performed by Bergiers at al [8], the assumption that edge-wear would develop at the superior acetabular edge (in vivo) had not been confirmed. Furthermore, no other method has allowed the investigation of the anterior-posterior distribution of edge-wear.

The subtly posterior location of the primary wear scar centre, measured from the mean acetabular model, was in contrast to the more anteriorly located edge-wear scars found in Bergiers et al.’s study [8]. This is likely due to the differences in approach, as the method adopted in the present study allowed the additional inclusion of non-edge worn implants, and its mean surface output was influenced by linear wear depth. Nevertheless, in agreement with their study [8], the distribution of volumetric wear in the mean model was skewed towards the anterosuperior quadrant. This observation is consistent with previously recorded hip joint forces that were directed anteriorly during a greater portion of walking gait [26,27], while posteriorly directed hip joint forces were only recorded during the heel-strike portion of the gait cycle [28]. This is supported by degradation patterns observed in acetabular cartilage, through both interoperative and MRI assessments [29,30,31,32]. Furthermore, these studies found at least some degree of degradation across a large portion of the superior cartilage structure, consistent with the mean acetabular wear identified in the present investigation. Several studies using instrumented implants with embedded force sensors have also identified greater amounts of pressure in these anterosuperior locations [33,34,35].

A similar relationship was observed between a greater degree of linear wear and a more anteriorly centred wear scar. This was evident within the two most dominant modes of variation, identified from the PCA analysis of the BHR acetabular surfaces. As the linear wear increased within the primary scar, its location was found to translate anteriorly. More anteriorly located wear scars were also concentrated to a narrower area, while posteriorly centred scars had a greater span towards the centre of the acetabular cup. This movement of wear patterns towards the acetabular centre could be associate with more optimal positioning, where the force vector would run through the centre of both the cup and head components [36]. This would result in a more central contact patch and reduced linear wear. The ability to assess the span of wear towards the pole in this manner could not be achieved using the approach adopted by Bergiers et al. [8], as it was restricted to wear found at the acetabular edge.

The population of acetabular surfaces included in this study was divided into contrasting subgroups based on their volumetric wear, time to revision, acetabular component positioning, gender and component size. This was conducted to isolate the influence of these SIP factors on the in vivo location acetabular wear. As expected, surfaces that exhibited volumes of material loss in excess of 2 mm^3^ had a more prominent edge-wear scar with greater linear depth. Consistent with the wear scar observed on the mean surface, its centre was located in the posterosuperior quadrant. This subgroup of implants was comparable to the population of implants assessed by Bergiers et al. [8], which again highlights the impact of greater linear wear on the mean acetabular surface. This may explain why the mean surface of low wearing components displayed edge-wear scars, despite the expectation for them to present more central and evenly distributed wear (unless damaged by rare cases of impingement) [8]. Another contributor to this trend may have been the contrast between edge-wear scars and other wear patterns, muting their depiction in the surface deviation maps.

The literature suggests that acetabular component positioning contributes to edge-loading and subsequently edge wear, particularly acetabular inclination. This is supported by the findings of this study, as BHRs positioned with greater inclination beyond Lewinnek’s safe zone had a larger and deeper primary wear scar located at the superior edge of the acetabular cup [19]. Greater inclination decreases the distance between the cup-head contact patch and the rim, especially in an implant such as the BHR, as it has a relatively large clearance (compared to other bearing designs) that increases the size of its bearing contact patch. 

The fact that the implants were within or outside this safe zone with respect to inclination, however, did not influence the anterior-posterior location of their wear scars, being centred about an angle of 8° and 7° into the posterosuperior quadrant, respectively. Contrastingly, the BHR implants that been positioned with an anteversion within Lewinnek’s safe zone were centred about the vertical CAPP axis (1°), which is considered representative of the vertical standing pelvic position. All but one of the acetabular cups that were outside this safe zone, had an anteversion greater than 25°. These surfaces had a mean wear scar located deep within the posterosuperior quadrant (−17°), which suggests a relationship between greater anteversion and more posteriorly located scars. As a larger proportion of the acetabular surfaces included in this study were positioned with high anteversion, it may also explain the more posteriorly positioned wear scar found on the mean surface generated from the entire population. Components with an anteversion within Lewinnek’s safe zone had a lager and deeper edge-wear scar. This was likely due to the influence of inclination on the development of wear on these cups, as the reader must be reminded that only four implants from this cohort were collectively well positioned, considering both inclination and anteversion. 

The BHRs revised less than 80 months after implantation were found to have a primary wear scar that spanned a greater portion of the superior acetabular edge and presented greater linear wear, compared to the implants that had remained in vivo for a longer duration. As greater volumes of material loss have been correlated with more severe cases of ARMD in periprosthetic tissue, this may explain why these implants were removed sooner [37,38]. This trend was also apparent in females and smaller cup sizes, which are considered closely associated. These are two further patient and implant factors that have been previously linked with elevated levels of material loss [25,39]. This is a result of the relationship found between smaller implants and a reduced arc of coverage angle, as it decreases the distance between the head-cup contact patch and the acetabular rim, increasing the probability of edge-loading [2,20,39,40].

Improving the statistical shape model generated in the present study would rely on a greater sample of acetabular surfaces. This would enhance the generalisability of the model, extending its representation to a larger proportion of the material loss volumes observed during retrieval analyses. This would have a particular impact on the comparisons made between subgroups of acetabular surfaces in this investigation, strengthening the statistical power of the observed trends. Future studies that iteratively investigate the output of the SSM as the input sample size is increased, will help define the appropriate number of samples that should be incorporated into a model such as this. Nevertheless, scarce is the opportunity to analyse 20 retrieved hip implants of a single design, which were accompanied with pre-revision, full pelvis medical CT data. This study could only be achieved due to the metal composition and asymmetric features of this specific design, which was needed to topographically visualise the acetabular component and orientate it within the patient. Although this investigation was reliant on the x-ray attenuation of MOM implants, the relevance of its findings extends far beyond the historic issues observed with this type of bearing design. The observed wear patterns could be generalised to better understand the bearing mechanics of all hip replacements, and even the native hip joint. 

The statistical shape approach detailed in this article could also be further augmented by tying the investigated SIP characteristics (i.e., wear volume, time to revision, etc.) directly to each acetabular surface when generating the model. This would allow their contribution to the variation in wear patterns to be better visualised, and should be undertaken in future research. Additionally, the authors acknowledge the limitations of adopting Lewinnek’s safe zone to evaluate component positioning, as it does not consider such factors as functional positioning during regular activities [8,41,42]; however, this method could contribute to a more reliable definition of optimal component positioning. We also recognise the potential for some variability to exist during the alignment of the acetabular cup within the pelvis model. This originates from the challenge of appropriate removal of metal artefacts and the clear identification of the stabilising fins and cup rim, separate from scanning noise. All analysis and measurements were performed by an experienced examiner however future studies should investigate the impact of any such variability that may exist in alignment. The influence of femoral anteversion, spine mobility and other patient factors were not investigated in this study, and their inclusion in future research would enhance our understanding of the development and location of hip implant wear. 

The further adoption of this method could provide a unique insight into the mechanics of hip replacements, no matter what their composition. Minimising material loss at the bearing surface remains a challenge for implant manufacturers, as seen by the continued use of hip simulators and the diversity of material combinations used to form this junction. Data provided by this method could contribute to this goal, informing the design of future implants. It could also inform surgeons of the range of component positions that lead to optimal wear performance, or highlight the narrow positioning window provided by specific implant designs. Moreover, it is certain that statistical shape modelling will continue to play a role in the examination of the human anatomy, while ever becoming an invaluable tool in the design and conception of orthopaedic implants.

## 5. Conclusions

This is the first study to apply statistical shape modelling to the analysis of retrieved orthopaedic implants. Its role within the presented method provides a unique insight into the mechanics of hip replacements, which can inform future implant design and the study of pathological anatomies. This SSM approach may also help to refine the safe zone for implant positioning. This study demonstrated that the mean wear patterns on the acetabular surface of these implants are located at their superior edge, and centred about the vertical standing pelvic position. The future implementation of this methodology to a larger population will further the current understanding of shape variance within orthopaedic implants retrieved from patients.

## Figures and Tables

**Table 1:** Criteria used to subdivide the population of BHR acetabular surfaces for comparison. The total sample size including in the final model was 19, following the exclusion of the outlier high wearing example.

Variable	Group 1	Group 2
Volume of Material Loss	<2 mm^3^_(n = 9)_	≥2 mm^3^_(n = 10)_
Inclination	30° ≤ x ≤ 50° _(n = 10)_	Any other Angle _(n = 9)_
Anteversion	5° ≤ x ≤ 25° _(n = 7)_	Any other Angle _(n = 12)_
Head Size	≤52 mm _(n = 10)_	>52 mm _(n = 9)_
Gender	F _(n = 12)_	M _(n = 7)_
Time to Revision	<80 months _(n = 9)_	≥80 months _(n = 10)_

## Data Availability

The data presented in this study are available on request from the corresponding author.

## References

[1] Bergiers S., Hothi H., Henckel J., Eskelinen A., Skinner J., Hart A. (2020). Does diametrical clearance influence the wear of Pinnacle hip implants?. Bone Jt. Res..

[2] Langton D.J., Jameson S.S., Joyce T.J., Hallab N.J., Natu S., Nargol A.V. (2010). Early failure of metal-on-metal bearings in hip resurfacing and large-diameter total hip replacement: A consequence of excess wear. J. Bone Jt. Surg.-Ser. B.

[3] Underwood R., Matthies A., Cann P., Skinner J.A., Hart A.J. (2011). A comparison of explanted Articular Surface Replacement and Birmingham Hip Resurfacing components. J. Bone Jt. Surg.-Ser. B.

[4] Park S.H., Lu Z., Hastings R.S., Campbell P.A., Ebramzadeh E. (2018). Five Hundred Fifty-five Retrieved Metal-on-metal Hip Replacements of a Single Design Show a Wide Range of Wear, Surface Features, and Histopathologic Reactions. Clin. Orthop. Relat. Res..

[5] Morlock M.M., Bishop N., Zustin J., Hahn M., Rüther W., Amling M. (2008). Modes of implant failure after hip resurfacing: Morphological and wear analysis of 267 retrieval specimens. J. Bone Jt. Surg. Am..

[6] Bills P.J., Racasan R., Underwood R.J., Cann P., Skinner J., Hart A.J., Jiang X., Blunt L. (2012). Volumetric wear assessment of retrieved metal-on-metal hip prostheses and the impact of measurement uncertainty. Wear.

[7] Hart A.J., Sabah S.A., Bandi A.S., Maggiore P., Tarassoli P., Sampson B., Skinner J.A. (2011). Sensitivity and specificity of blood cobalt and chromium metal ions for predicting failure of metal-on-metal hip replacement. J. Bone Jt. Surg.-Ser. B.

[8] Bergiers S., Hothi H., Henckel J., Di Laura A., Belzunce M., Skinner J., Hart A. (2021). The in vivo location of edge-wear in hip arthroplasties: Combining pre-revision 3D CT imaging with retrieval analysis. Bone Jt. Res..

[9] Barratt D.C., Chan C.S.K., Edwards P.J., Penney G.P., Slomczykowski M., Carter T.J., Hawkes D.J. (2008). Instantiation and registration of statistical shape models of the femur and pelvis using 3D ultrasound imaging. Med. Image Anal..

[10] Meynen A., Vles G., Roussot M., Van Eemeren A., Wafa H., Mulier M., Scheys L. (2022). Advanced quantitative 3D imaging improves the reliability of the classification of acetabular defects. Arch. Orthop. Trauma Surg..

[11] Meynen A., Matthews H., Nauwelaers N., Claes P., Mulier M., Scheys L. (2020). Accurate reconstructions of pelvic defects and discontinuities using statistical shape models. Comput. Methods Biomech. Biomed. Engin..

[12] Hettich G., Schierjott R.A., Ramm H., Graichen H., Jansson V., Rudert M., Traina F., Grupp T.M. (2019). Method for quantitative assessment of acetabular bone defects. J. Orthop. Res..

[13] Vanden Berghe P., Demol J., Gelaude F., Vander Sloten J. (2017). Virtual anatomical reconstruction of large acetabular bone defects using a statistical shape model. Comput. Methods Biomech. Biomed. Engin..

[14] (2016). Implants for Surgery—Wear of Total Hip-Joint Prostheses—Part 2: Methods of measurement Implants.

[15] (2014). Standard Guide for Characterization of Wear from the Articulating Surfaces in Retrieved Metal-on-Metal and other Hard-on-Hard Hip. www.astm.org.

[16] Bergiers S., Hothi H., Richards R., Henckel J., Hart A. (2019). Quantifying the bearing surface wear of retrieved hip replacements. Biosurf. Biotribol..

[17] Bergiers S., Hothi H., Richards R., Dall’Ava L., Henckel J., Hart A. (2020). Quantifying material loss from the bearing surfaces of retrieved hip replacements: Method validation. Tribol. Int..

[18] Amstutz H., Jacobs J., Ebramzadeh E. (2011). Current Status of Metal-on-Metal Hip Resurfacing, an Issue of Orthopedic Clinics.

[19] Lewinnek G.E., Lewis J.L., Tarr R., Compere C.L., Zimmerman J.R. (1978). Dislocations after total hip-replacement arthroplasties. J. Bone Jt. Surg.

[20] Campbell P., Beaulé P.E., Ebramzadeh E., Le Duff M.J., De Smet K., Lu Z., Amstutz H.C. (2006). The john charnley award: A study of implant failure in metal-on-metal surface arthroplasties. Clin. Orthop. Relat. Res..

[21] Ebramzadeh E., Campbell P.A., Takamura K.M., Lu Z., Sangiorgio S.N., Kalma J.J., De Smet K.A., Amstutz H.C. (2011). Failure Modes of 433 Metal-on-Metal Hip Implants: How, Why, and Wear. Orthop. Clin. N. Am..

[22] Bergiers S., Hothi H.S., Henckel J., Eskelinen A., Skinner J., Hart A. (2018). Wear performance of retrieved metal-on-metal Pinnacle hip arthroplasties implanted before and after 2007. Bone Jt. Res..

[23] De Haan R., Campbell P.A., Su E.P., De Smet K.A. (2008). Revision of metal-on-metal resurfacing arthroplasty of the hip: The influence of malpositioning of the components. J. Bone Jt. Surg.-Ser. B.

[24] Kwon Y.M., Glyn-Jones S., Simpson D.J., Kamali A., McLardy-Smith P., Gill H.S., Murray D.W. (2010). Analysis of wear of retrieved metal-on-metal hip resurfacing implants revised due to pseudotumours. J. Bone Jt. Surg.-Ser. B.

[25] Underwood R.J., Zografos A., Sayles R.S., Hart A., Cann P. (2012). Edge loading in metal-on-metal hips: Low clearance is a new risk factor. Proc. Inst. Mech. Eng. Part H J. Eng. Med..

[26] Correa T.A., Crossley K.M., Kim H.J., Pandy M.G. (2010). Contributions of individual muscles to hip joint contact force in normal walking. J. Biomech..

[27] Lewis C.L., Sahrmann S.A., Moran D.W. (2008). Effect of Hip Angle on Anterior Hip Joint Force during Gait. Gait Posture.

[28] Uddin M.S., Zhang L.C. (2013). Predicting the wear of hard-on-hard hip joint prostheses. Wear.

[29] Tannast M., Goricki D., Beck M., Murphy S.B., Siebenrock K.A. (2008). Hip damage occurs at the zone of femoroacetabular impingement. Clin. Orthop. Relat. Res..

[30] Shibata K.R., Matsuda S., Safran M.R. (2017). Is there a distinct pattern to the acetabular labrum and articular cartilage damage in the non-dysplastic hip with instability?. Knee Surg. Sport. Traumatol. Arthrosc..

[31] Hingsammer A., Chan J., Kalish L.A., Mamisch T.C., Kim Y.J. (2013). Is the damage of cartilage a global or localized phenomenon in hip dysplasia, measured by dGEMRIC?. Hip. Clin. Orthop. Relat. Res..

[32] Zilkens C., Tiderius C.J., Krauspe R., Bittersohl B. (2015). Current knowledge and importance of dGEMRIC techniques in diagnosis of hip joint diseases. Skelet. Radiol..

[33] Davy D.T., Kotzar G.M., Brown R.H., Heiple K.G., Goldberg V.M., Heiple K.G., Berilla J., Burstein A.H. (2009). Telemetric force measurements across the hip after total arthroplasty Telemetric across Measurements Total the Hip after. Surgery.

[34] Bergmann G., Deuretzbacher G., Heller M., Graichen F., Rohlmann A., Strauss J., Duda G.N. (2001). Hip contact forces and gait patterns from routine activities. J. Biomech..

[35] Hodge W.A., Carlson K.L., Fijan R.S., Burgess R.G., Riley P.O., Harris W.H., Mann R.W. (1989). Contact pressures from an instrumented hip endoprosthesis. J. Bone Jt. Surg.-Ser. A.

[36] Fisher J. (2011). Bioengineering reasons for the failure of metal-on-metal hip prostheses. J. Bone Jt. Surg. Br..

[37] Mahendra G., Pandit H., Kliskey K., Murray D., Gill H.S., Athanasou N. (2009). Necrotic and inflammatory changes in metal-on-metal resurfacing hip arthroplasties: Relation to implant failure and pseudotumor formation. Acta Orthop..

[38] Haddad F.S., Thakrar R.R., Hart A.J., Skinner J.A., Nargol A.V., Nolan J.F., Gill H.S., Murray D.W., Blom A.W., Case C.P. (2011). Metal-on-metal bearings: The evidence so far. J. Bone Jt. Surg.-Ser. B.

[39] Langton D.J., Jameson S.S., Joyce T.J., Webb J., Nargol A.V. (2008). The effect of component size and orientation on the concentrations of metal ions after resurfacing arthroplasty of the hip. J. Bone Jt. Surg.-Ser. B.

[40] Langton D.J., Joyce T.J., Jameson S.S., Lord J., Van Orsouw M., Holland J.P., Nargol A.V., De Smet K.A. (2011). Adverse reaction to metal debris following hip resurfacing: The influence of component type, orientation and volumetric wear. J. Bone Jt. Surg.-Ser. B.

[41] Tezuka T., Heckmann N.D., Bodner R.J., Dorr L.D. (2019). Functional Safe Zone Is Superior to the Lewinnek Safe Zone for Total Hip Arthroplasty: Why the Lewinnek Safe Zone Is Not Always Predictive of Stability. J. Arthroplast..

[42] Dorr L.D., Callaghan J.J. (2019). Death of the Lewinnek “Safe Zone”. J. Arthroplast..

